# Evaluation of Preincubation Time Interval in Testicular Biopsy to Obtain Optimum Sperm Parameters

**Published:** 2012-06-13

**Authors:** Yousef-Reza Yousefnia Pasha, Seyed Gholam-Ali Jorsaraei, Mahtab Zeinalzadeh, Masoumeh Golsorkhtabar Amiri, Arsalan-Ali Ramaji

**Affiliations:** 1. Fatemeh-Zahra Infertility and Reproductive Health Research Center, Babol University of Medical Sciences, Babol, Iran; 2. Department of Urology , Shahid Beheshti Hospital, Babol University of Medical Sciences, Babol, Iran

**Keywords:** Preincubation Time, Sperm Parameters, Testicular Biopsy

## Abstract

**Objective:**

In sever oligospermia; one of the paths used for surgical sperm retrieval (SSR) is to extract sperm via a testicular biopsy. The aim of our study is to determine the reliable time interval between testicular biopsy and intracytoplasmic sperm injection (ICSI) procedure in order to obtain optimumsperm parameters (count, motility and normal morphology).

**Materials and Methods:**

This cohort study was carried out on 30 patients which were candidates for ICSI. After collection and keeping the samples obtained from the testicular biopsy in Ham’s F10 environment, the concentration, motility and morphology of the sperm in each sample was evaluated immediately as well as 2 and 4 hours after processing. The Data were then compared with each other. For the statistical analysis, Friedman, Willcoxon and Cochran tests were used.

**Results:**

The mean of sperm concentration was 5.69 ± 6.14 million and the motility was10.83 ± 12.63% at 2 hours following biopsy which was significantly higher than those obtained after 0 and 4 hours of the biopsy (p <0.05).

**Conclusion:**

The reliable preincubation time which resulted in the highest rate of spermatozoa parameters after testicular biopsy and before incubation was 2 hours.

## Introduction

Oligospermia is a common cause of male infertility. Fortunately, for many sub infertile men, can be treated within weeks simply by making lifestyle changes. Other men may require fertility treatment to increase their chances of conceiving a child. Assisted reproductive technique (ART) in surgical sperm retrieval (SSR) helps reproductive urologists to extract sperm from the vas deferens, epididymis or directly from testis in men without sperm or with a few sperm present in their semen sample (1). The testicular sperm are mostly immotile immediately after biopsy and in particular after thawing of frozen testicular samples (2). To avoid, the suspension of testicular cells the sample is incubated in the culture medium (Ham’s F10) for a determined period of time. There is a study of obstructive azoospermia which consists of improving sperm motility of fresh samples by culturing them in the medium for 3 days, before intracytoplasmic sperm injection (ICSI) or freezing. This would allow the biopsy to be taken 2-3 days before oocyte retrieval which would ease the selection of motile spermatozoa for ICSI (3). In a similar study on oocyte, Bahiraee et al. compared the effect of time of partenogenetic activation [22 hours vs 27 hours after *in vitro* maturation (IVM)] on subsequent development of IVM-derived bovine oocytes using either single activation agents (ionomycin 5 µM for 5 minutes) or combined activation treatments (ionomycin with 6-DMAP 2.0 mM for 3 hours). They concluded6-DMAP 2.0 mM for 3 hours). They concludedcoded that blastocyst formation, like cleavage formation, significantly increased when the maturation time was increased from 22 hours to 27 hours (4). With ICSI, there remains debate among embryologists regarding the time interval between semen processing and incubation as an effective factor on sperm parameters and outcome of ICSI. However, another study was conducted which showed there was no such effect on *in vitro* fertilization (IVF) (5). Delays of up to 15 hours in processing and cryopreservation of a testicular biopsy did not affect the viability of the extracted sperm after thawing in ICSI (6). Although it is desirable to freeze the testicular specimen as soon as possible, the delay in processing in this case due to keep in outside of laboratory environment did not affect the potential for fertilization and implantation of a viable cryopreserved blastocyst (6).

A query reported increasing spermatozoa preincubation time may improve sperm parameters and also, incubation of freezing sperm obtained of testicular biopsy may improve sperm motility (3, 7). Other researchers have reported that the acrosome reaction occurs spontaneously during incubation in a defined medium (7-9) and therefore differ at various times (10). In a study, the optimum incubation time of spermatozoa before ICSI which resulted in the highest fertilization rate, was stated to be 3 hours (8). Also, recently several studies have examined the effect of different oocyte preincubation time on fertilization, cleavage, and implantation rates (11- 13). In testicular sperm obtaining, increase vital sperm, enhance fertilization rates and increase the number of embryos are desirable for all ICSI laboratories. The objective of our study is to determine the optimum time interval between testicular biopsy and ICSI in order to obtain desirable sperm parameters.

## Materials and Methods

This study was performed in 2009 on 30 patients undergoing ICSI in Fatemeh-Zahra Infertility and Reproductive Health Research Center, (North of Iran)and has been approved by the Ethical Committee of Babol University of Medical Science. All 30 patients signed a written informed consent form. The subjected patients were under 40, who had undergone surgical retrieval of sperm required for ICSI. Also, all the patients were oligoasthenoteratospermia with normal hormonal assay.

In this study patients with no sperm in biopsy, severe sport injuries or testis trauma, those undergoing radiotherapy or chemotherapy as well as those addicted to drugs or alcohol were excluded 2 months prior to the study.

### Spermatozoa preparation

About 7-10 cc lidocaine 2% was injected within a testicular cord and the incision was 2-3 cm on the side of scrotal skin. The testis was exposed following the incisions of the skin, subcutaneous tissue and Tunica vaginalis. Then, a little testis tissue was biopsied and processed in ART lab. The incisions were repaired with 0-2 chromic cotton. It should be noted that the biopsied sample was placed in a dish with an environment consisting of 10% Ham’s F10, which was prepared by adding 1cc of albumin to 9 cc Ham’s F10. The tubules within the biopsied sample were totally opened by a special needle. Potentially, any existing sperm cells should enter the cell culture media when the tissue is broken in this way. The sperm cells should then be pulled by pipette along with the media and a drop of this solution should be placed onto a microscopic slide which is then analyzed under the microscope in order to detect the presence or absence of sperm within the sample. The remnant of the mentioned mixture was centrifuged at 500 rpm allowing the pieces of tissue to sink to the bottom of the dish, the mixture was then centrifuged again at 3000 rpm for 5 minutes and then sperm were placed in a more transparent environment. The liquid on the top of tube was then discarded and 0.5 cc of media was added to the sediments at the bottom of the tube. Then by shaking the tube, a uniform sample was obtained and a drop of the resultant solution was placed on the slide of microscope. The slides were then analyzed under the microscope and the concentration, motility and morphology of the spermatozoa present in each sample was evaluated immediately after processing, as well as 2 and 4 hours following processing. The data was analyzed using SPSS16 and Fridman, Willcoxon and Cochran tests were conducted. A p<0.05 was considered significant.

## Results

Thirty patients entered to this study. Demographic criteria were shown in table1.

**Fig 1:**
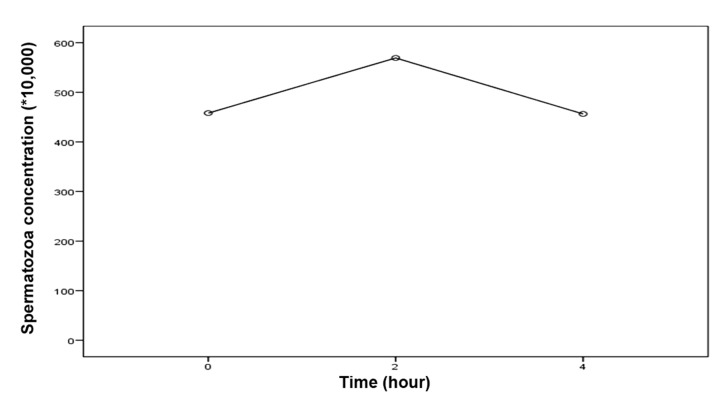
Rate of spermatozoa concentration in various hours after biopsy.

**Fig 2:**
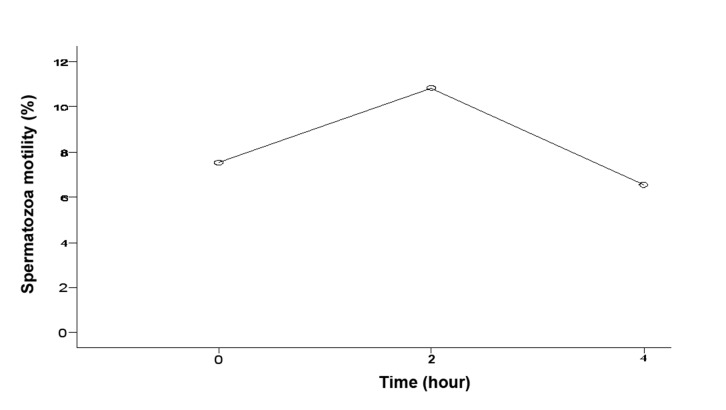
Rate of spermatozoa motility in various hours after biopsy.

**Fig 3:**
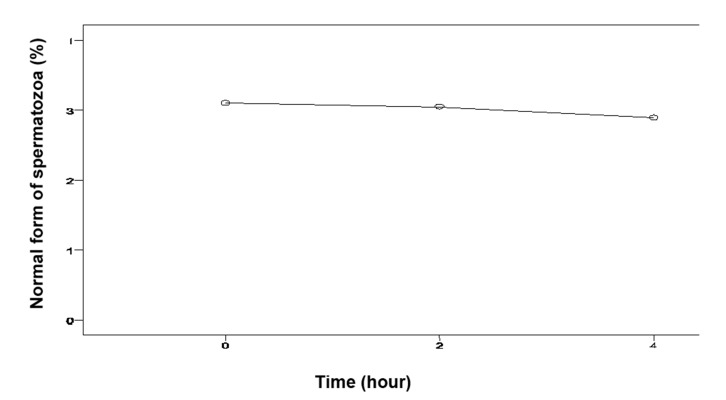
Rate of normal spermatozoa in various hours after biopsy.

The mean of sperm concentration was 5.69 ± 6.14 millions and the motility was 10.83 ± 12.63% 2 hours after the biopsy. These results were significantly higher than the results obtained from sperm which were analyzed immediately after the biopsy and 4 hours after biopsy (p<0.05, Table 2). All the results are summarized in the tables (Figs 1- 3).

**Table 1:** Demographic criteria of patients undergone testis biopsy


Age (year)	32.71 ± 0.79
Infertility duration (year)	1.990 ± 0.53


**Table 2:** Sperm parameters in various hours following testis biopsy


Sperm parameters	Time (hours)	Mean ± SE

Concentration (104)	0	458.02 ± 60.12
	2	569.24 ± 80.04 ^a^
	4	456.34 ± 70.46 ^b^
Motility (%)	0	7.53 ± 1.36
	2	10.83 ± 1.64 ^a^
	4	6.54 ± 1.29 ^b^
Normal form (%)	0	3.10 ± 0.39
	2	3.05 ± 0.36
	4	2.90 ± 0.33


a: Significant at p<0.05 when compared to data of time 0. b: Significant at p<0.05 when compared to data of time 2.

## Discussion

In this study, the reliable time interval between testicular biopsy and injecting the spermatozoa in to the oocyte resulting in the extraction of sperm with the highest parameters was suggested to be 2 hours. In most IVF centers, this time interval is dependent on the workload of the ART Laboratory. The embryologist can easily distinguish and extract spermatozoa during the testicular biopsy, but they are mostly immotile and it is the use of motile sperm which significantly increases the efficacy of the ICSI procedure (3, 14).

To improve sperm motility, Morrisa suggested incubation in processing media or Ham’s F10 media between 24 and 48 hours in the case of patients with Azospermia. In samples obtained from patients with obstructive Azospermia, the motility at 1, 24 and 48 hours was 3%, 20% and 25% respectively. In the samples obtained from patients with non-obstractive Azospermia, the motility at 1, 24 and 48 hours was zero, 5% and 11%, respectively (7).

A study conducted by Windt et al. determined the number of motile sperm in testicular tissue. Their results showed that sperm motility was enhanced by pre-incubation in culture medium (15). Other researchers observed successful outcome in ICSI using sperm from testicular tissue that started moving after prolonged cultivation in culture media (3, 16) or using the hypo-osmotic swelling (HOS) test in cases of total immobility (17). It appears that both of these tests may be harmful for sperm parameters. However, some studies have shown that long-term exposure of oocytes to sperm may be harmful for fertilization (8, 18).

There is a debate about whether or not the probability of the resulting embryos to implant may be dependent on the sperm injection time (19, 9). In previous studies (20, 21), an optimal IVF outcome is obtained by the insemination of oocytes after a pre-incubation period of 3-5.5 hours time after sperm retrieval (20, 21). Moreover, Rienzi et al. showed that a preincubation interval time of at least 3 hour between oocyte retrieval and injection in ICSI could enhance the fertilization rate (22). In another study, Movahedin et al. reported that the sperm parameters improved with time (23). Our study was comparable to these studies; however, there are several studies which disagree with our results. Coskun et al. implied that the results of fertilization and embryo development were comparable when insemination was performed after 1hour of retrieval and after 18-hours (24). Van de Velde et al. (13) and Yanagrida et al. (12) reported the timing of injection had no significant effects on ICSI fertilization and pregnancy rate There is another study by Jacobs et al. in which 881 *IVF* and 432 ICSI cycles insemination or injection was done 1-7 or 0.5-8 hours following oocyte retrieval and concluded that no significant differences were found between the time and outcome of ICSI and IVF regarding fertilization rates (25). It is interesting to refer to a study conducted by wramsby et al. which explained that spermatozoa of the samples were pre-incubated 4 or 24 hours before ova were inseminated during 3 hours. An extended insemination for an additional 3 hours was also done after the shorter pre-incubation period. They concluded that a combination of short and long pre-incubation periods should be applied to raise the success of fertilization (26).

## Conclusion

Our study showed that the sperm parameter is at its optimum rate 2 hours following testicular biopsy. Therefore, we recommend a 2-hour gap between obtaining sperm from the testis and using them to perform ICSI.
